# The Culture Dish Surface Influences the Phenotype and Dissociation Strategy in Distinct Mouse Macrophage Populations

**DOI:** 10.3389/fimmu.2022.920232

**Published:** 2022-07-06

**Authors:** Qiaoling Song, Yazhuo Zhang, Mingming Zhou, Yuting Xu, Qianyue Zhang, Lihong Wu, Shan Liu, Minghui Zhang, Lei Zhang, Zhihua Wu, Weixun Peng, Xutao Liu, Chenyang Zhao

**Affiliations:** ^1^ School of Medicine and Pharmacy, Ocean University of China, Qingdao, China; ^2^ Innovation Platform of Marine Drug Screening and Evaluation, Qingdao National Laboratory for Marine Science and Technology, Qingdao, China; ^3^ College of Food Science and Engineering, Ocean University of China, Qingdao, China; ^4^ Faculty of Medicine, Imperial College London, London, United Kingdom; ^5^ Samueli School of Engineering, University of California Los Angeles, Los Angeles, CA, United States

**Keywords:** dissociation methods, culture dish surface, macrophage polarization, transcriptome sequencing, LPS challenge

## Abstract

The nature of the culture dish surface and the technique used to detach adherent cells could very likely influence the cell viability and cell membrane protein integrity of harvested macrophages. Several previous studies assessed the detachment efficacies of enzymatic and non-enzymatic methods for harvesting the single cell suspensions of macrophages, but a comprehensive study assessing different dissociation methods and culture conditions for detaching functionally different macrophage populations has not yet been reported. In this study, *via* the well-established GM-CSF and M-CSF differentiated bone marrow derived macrophage models (GM-BMDMs and M-BMDMs), we compared four commonly used enzymatic (trypsin and accutase) and non-enzymatic (PBS and EDTA) dissociation methods along with necessary mechanical detaching steps (scraping and pipetting) to evaluate the viable cell recovery and cell surface marker integrality of GM-BMDMs and M-BMDMs cultured on standard cell culture dish (TC dish), or on culture dish (noTC dish) that was not conditioned to enhance adherence. The data showed that accutase yielded a better recovery of viable cells comparing with PBS and EDTA, especially for tightly adherent GM-BMDMs on TC dishes, with a relatively higher level of detected cell membrane marker F4/80 than trypsin. An additional gradient centrifugation-based dead cell removal approach could increase the proportion of viable cells for TC cultured GM-BMDMs after accutase dissociation. Furthermore, transcriptome analysis was performed to evaluate the putative influence of culture dishes. At steady state, BMDMs cultured on noTC dishes exhibited more proinflammatory gene expression signatures (e.g. IL6, CXCL2 and ILlβ) and functions (e.g. TNF and IL17 signaling pathways). Similar inflammatory responses were observed upon LPS challenge regardless of culture conditions and differentiation factors. However, in LPS treated samples, the difference of gene expression patterns, signaling pathways and molecular functions between TC and noTC cultured BMDMs were largely dependent on the types of growth factors (M-CSF and GM-CSF). This observation might provide valuable information for *in vitro* macrophage studies.

## Introduction

The use of bone marrow derived macrophages (BMDMs) is one of the most well-established means of studying macrophage biology *in vitro*, as they were believed to represent the phenotypes and characteristics of *in vivo* macrophages (1). GM-CSF and M-CSF derived BMDMs (GM-BMDMs and M-BMDMs) could resemble different macrophage maturation features of classically activated proinflammatory M1-like and alternatively activated restorative M2-like macrophages, respectively (2). Both membrane protein integrity and cell viability during dissociation are critical for successful downstream biological studies (3, 4), including flow cytometry and single cell RNA sequencing. Besides, cell adhesion characteristics vary with macrophage maturation/polarization (5, 6). Therefore, it is essential to determine the appropriate dissociation methods for different macrophage populations.

Multiple attempts were made to optimize the cell culture condition and methods to diassociate M-CSF differentiated macrophages. Replacing standard tissue culture dishes treated to promote adherence (TC, hydrophilic, facilitating cell attachment) with untreated dishes (noTC, hydrophobic, reducing cell adhesion), or utilizing specific dissociation conditions, including nonenzymatic (EDTA or PBS solutions) or enzymatic ones (accutase or trypsin solutions), are the conditions typically employed to harvest the single cell suspension of BMDMs. In previous studies, accutase was reported to be a mild and highly efficient dissociation solution for detaching M-CSF primed monocyte derived macrophages (M0), IL4 challenged M0 (M2) and LPS+IFNγ challenged M0 (M1) when compared with EDTA and trypsin solutions (7, 8). However, the efficiency of different dissociation methods for BMDMs differentiated by GM-CSF, a growth factor previously proven to enhance the adhesion of monocytes, neutrophils, and macrophages (9–11), has not been reported, nor has there been a comparison made between the ease of dissociating GM-BMDMs and M-BMDMs. It is worthwhile to determine the optimal solution and condition for dissociating both GM- and M-BMDMs, since these two types of BMDMs are usually compared for their signaling transduction pathways, macrophage function and gene expression patterns.

TC and noTC dishes have been indiscriminately used in most BMDM related studies *in vitro* even though the anchorage capacities were theoretically weaker for noTC dishes. With both dishes, some BMDMs’ characteristics were studied and considered to be not influenced by culture conditions. For example, TC cultured BMDMs were utilized to explore the immunocompetence of GM-CSF and M-CSF primed macrophages and the heterogeneity of cell populations in GM-CSF primed bone marrow cells (12, 13). The noTC dish cultured BMDMs were used to analyze gene expression patterns of GM-BMDMs and M-BMDMs in microarray experiments and the secretion profiles at the single cell level (6, 14). The cell metabolism and epigenetic genome modification of BMDMs were studied without specifying the dish conditions (15, 16). Nevertheless, the impact of dish surface conditions for monocyte-derived dendritic cells has been reported (17). At present, the influence of TC and noTC culture conditions on surface marker expression and inflammatory responses of GM-CSF and M-CSF primed BMDMs are not yet thoroughly investigated, which makes interpreting results extremely difficult. Therefore, it is necessary to characterize the phenotypes of BMDMs under different culture conditions to determine whether there are biologically significant differences in terms of gene expression profile and cell functions.

In the present study, we applied two types of culture dishes (TC and noTC) for GM- and M-BMDMs maturation and used four different dissociation techniques to detach these BMDMs. As previously reported (18), accutase was found to be a mild and highly efficient dissociation reagent for all tested BMDMs, especially for the TC cultured GM-BMDMs that had the highest adhesive capacity. A gradient centrifugation-based dead cell removal procedure was additionally proposed as an extra step following accutase dissociation for the tightly adherent TC cultured GM-BMDMs in order to obtain more viable cells. Transcriptome analysis was also performed to determine whether culture conditions influence gene expression profiles of two types of BMDMs on TC and noTC culture dishes at both steady state and upon LPS treatment. The data suggested that at steady state, low adhesion on noTC culture dishes renders both GM-BMDMs and M-BMDMs more proinflammatory. LPS could induce MyD88/NFκB and TRIF/IRF signaling in all the tested BMDMs; however, under the stimulation of LPS, inflammatory responses and cell adhesion activities were upregualted in noTC-GM-BMDMs, whereas cell proliferation and metabolism functions were enriched in noTC-M-BMDMs. Together, our data reveal the culture dish surface-dependent signaling changes and provide the optimal dissociation method for TC and noTC cultured BMDMs for further functional studies.

## Material and Methods

### Antibodies and Reagents

TC treated cell culture dishes (Cat. F611203) and noTC treated polystyrene culture dishes (Cat. F611003) were purchased from Sangon Biotech (Shanghai). SYBR Green PCR Master Mix (2×) (Cat. 4913914001) was from Roche. RNAiso Plus (Cat. 9109) and PrimeScript™ RT reagent kit (Cat. RR047A) with gDNA eraser were from TaKaRa. LPS (Cat. L2630) and accutase solution containing 0.5 mM EDTA (Cat. A6964) were obtained from Sigma-Aldrich. 0.5 M EDTA was obtained from Solarbio (Cat. 420404). 0.25% Trypsin containing 0.02% EDTA was from Zhejiang Senrui Biological Technology (Cat.CR-25200). Recombinant mouse M-CSF (Cat. 415-ML) and GM-CSF (Cat. 416-ML) were purchased from R&D Systems. Debris Removal Solution (Cat. 130-110-203) and antibodies against Ly6C (APC) (Cat. 130-111-779) were from Miltenyi Biotec. 7-AAD Viability Staining Solution (Cat. 51-68981E) and antibodies against F4/80 (PE) (Cat. 565410) were from BD Bioscience. Anti-MHCII (Brilliant Violet 421) (Cat. 107632) antibodies were from Biolegend. Antibodies against CD11b (PE-CY7) (Cat. 25-0112-82) and CD16/CD32 (Cat. 14-0161-86) and Rat IgG1 kappa isotype control (Cat. 14-4301-85) were from Invitrogen.

### Animals

C57BL/6J mice (male, SPF degree, 6-10 weeks old) were purchased from Beijing Vital River Laboratory Animal Technology and maintained in a temperature- and humidity-controlled room with a 12 h light-dark cycle. All animal procedures were approved by the Committee of Experimental Animals of School of Medicine and Pharmacy, Ocean University of China (OUCSMP-20181002).

### Bone Marrow Derived Macrophage (BMDM) Culture

Mouse BMDMs were prepared as previously described (19). Briefly, bone marrow cells were collected from femurs. The cell suspensions were passed through 100 μm cell strainer, collected by centrifugation at 300 g for 10 minutes, and resuspended in DMEM containing 10% FBS, 100 U/ml penicillin, 100 μg/ml streptomycin, either with 50 ng/ml M-CSF or 20 ng/ml GM-CSF. Cells (0.5×10^6^ per ml for M-CSF and 1×10^6^ per ml for GM-CSF) were seeded on TC or noTC dishes respectively and cultured at 37°C with 5% CO2 for 7 days without changing the media throughout the maturation process. For RT-PCR and RNA-seq analysis, mouse BMDMs cultured on TC and noTC dishes were treated with vehicle or LPS (100 ng/ml) for 3 hrs.

### Cell Detachment and Dead Cell Removal

For the detachment of M-BMDMs cultured on TC (M-TC) and noTC (M-noTC) dishes and GM-BMDMs on noTC dishes (GM-noTC), cells were washed with PBS and incubated with trypsin or accutase at room temperature (RT) for the indicated times as shown in **Table 1**. Cells were pipetted without scraping and harvested. For tightly adherent GM-BMDMs cultured on TC dishes (GM-TC), cells were washed, incubated with trypsin or accutase at RT followed by further incubation at 37°C and scraped gently with cell lifters (Biologix, Cat. 70-2180) to harvest cell suspension. For nonenzymatic approaches, all these four types of BMDMs were washed and incubated with EDTA or PBS on ice for the indicated time with necessary mechanical steps (pipetting or scraping) (details in **Table 1**). DMEM media with 10% FBS were added to stop the digestion process. Cells were centrifuged at 300 g for 10 min, washed with PBS containing 2% FBS and processed for the indicated experiments.

**Table 1:** The comparisons of dissociation methods for BMDMs cultured on TC and noTC dishes.

Groups	GM-noTC			GM-TC^a^			M-noTC			M-TC		
	Time (min)	Temp	Scrape	Time (min)	Temp	Scrape	Time (min)	Temp	Scrape	Time (min)	Temp	Scrape
**Accutase**	**5**	**RT**	**-**	**5+10**	**RT+37**°C	**++**	**3**	**RT**	**-**	**5**	**RT**	**-**
**Trypsin**	**5**	**RT**	**-**	**5+10**	**RT+37**°C	**++**	**3**	**RT**	**-**	**5**	**RT**	**-**
**EDTA**	**15**	**on ice**	**+**	**15**	**on ice**	**+++**	**10**	**on ice**	**-**	**15**	**on ice**	**-**
**PBS**	**15**	**on ice**	**+++**	**15**	**on ice**	**+++**	**15**	**on ice**	**-**	**15**	**on ice**	**+++**

GM: GM-BMDMs, M: M-BMDMs, Temp: Temperature, Scrape: -, no scraping but pipetting; +, weak scraping, only a small proportion of cells need to be scraped after gently pipetting; ++, middle scraping, about half of cells need to be scraped after gently pipetting; +++, strong scraping, most of cells need to be scraped after gently pipetting

a for GM-TC samples, cells were digested with accutase or trypsin for 5 min at RT followed by additional incubation for 10 min at 37°C.

Density centrifugation for GM-TC samples was performed following manufacturer’s instructions (Debris removal solution, Miltenyi Biotec, Cat. 130-109-398). Briefly, detached macrophages were resuspended with 3.1 ml cold PBS and mixed well with 0.9 ml cold debris removal solution using 5 ml pipette in a 15 ml centrifuge tube. Additional 4 ml cold PBS was overlayed very gently. After centrifugation at 4°C at 3000 g for 10 min with full acceleration and no brake, three phases were formed and the top two layers were completely aspirated and discarded. After that, cold PBS were added to fill up the 15 ml tube, followed by gently mixing and centrifugation at 4°C at 1000 g for 10 min with full acceleration and brake. The supernatant was completely discarded and macrophages were resuspended for further experiments.

### Flow Cytometry

Cells were dissociated, washed and resuspended in blocking buffer (20% FBS, 1:100 CD16/CD32 antibodies and 1:100 Rat IgG1 isotype) for 30 min at 4°C. The fluorescence conjugated primary antibodies were added and then samples were washed. Subsequently, cells were stained with 7AAD buffer and acquired by FACS Aria III cytometer. All analyses were performed using FlowJo software.

### Reverse Transcription and Real-Time PCR

Mouse BMDMs cultured on TC and noTC dishes were treated with vehicle or LPS (100 ng/ml) for 3 hrs. Total RNA from cultured BMDMs was extracted with RNAiso Plus. Reverse transcription was performed with a PrimeScript™ RT reagent kit containing gDNA Eraser. The cDNA samples were amplified using SYBR Green in StepOne Plus Real-Time PCR System (Applied Biosystems). The relative mRNA expression was determined using the ΔCt relative quantification method, which means relative mRNA levels of target gene = 2ˆ(-ΔCt) = 2ˆ(-(CT_target gene_ - CT_reference gene_)). Specific primers used in the real-time PCR (RT-PCR) were as follow:

TNFα Forward: CAGGCGGTGCCTATGTCTC; TNFα Reverse:

CGATCACCCCGAAGTTCAGTAG; IL6 Forward:

TAGTCCTTCCTACCCCAATTTCC;IL6 Reverse:

TTGGTCCTTAGCCACTCCTTC;IL1β Forward:

GAAATGCCACCTTTTGACAGTG;IL1β Reverse:

TGGATGCTCTCATCAGGACAG;GAPDH Forward:

ATGCCTGCTTCACCACCTTCT;GAPDH Reverse:

CATGGCCTTCCGTGTTCCTA.

### Transcriptome Sequencing (RNA-Seq Analysis)

Mouse BMDMs cultured on the TC and noTC dishes were treated with vehicle or LPS (100 ng/ml) for 3 hrs. Total RNA was extracted using Trizol reagent kit (Invitrogen) according to the manufacturer’s protocol. The mRNA was enriched by Oligo (dT) beads, fragmented using fragmentation buffer and reversely transcribed into cDNA by using NEBNext Ultra RNA Library Prep Kit for Illumina (NEB #7530). The resulting cDNA library was sequenced using Illumina Novaseq6000 by Gene Denovo Biotechnology Co. Clean reads were aligned to the mouse reference genome using HISAT2.2.4 (20). For each transcription region, a FPKM value was calculated to quantify its expression abundance, using RSEM software (21). Differentially expressed genes (DEGs) were identified using the DESeq2 (22). P-value <0.05 and fold change >2 or 1.5 were set as the threshold for significantly differential expression. KEGG pathway (23), GO enrichment (24), Reactome analysis (25, 26), PPI (27) and IPA (28) analysis of DEGs were respectively performed. The scaled FPKM values were normalized using z-score approach (z = (x-μ)/σ), where x is the FPKM value, μ is the mean of FPKM values of certain gene in all tested samples (row mean), and σ is the standard deviation of row values (29). The RNAseq data were deposited in the Gene Expression Omnibus (GEO) database under the accession number GSE198821.

### Statistical Analysis

**Figure 1:**
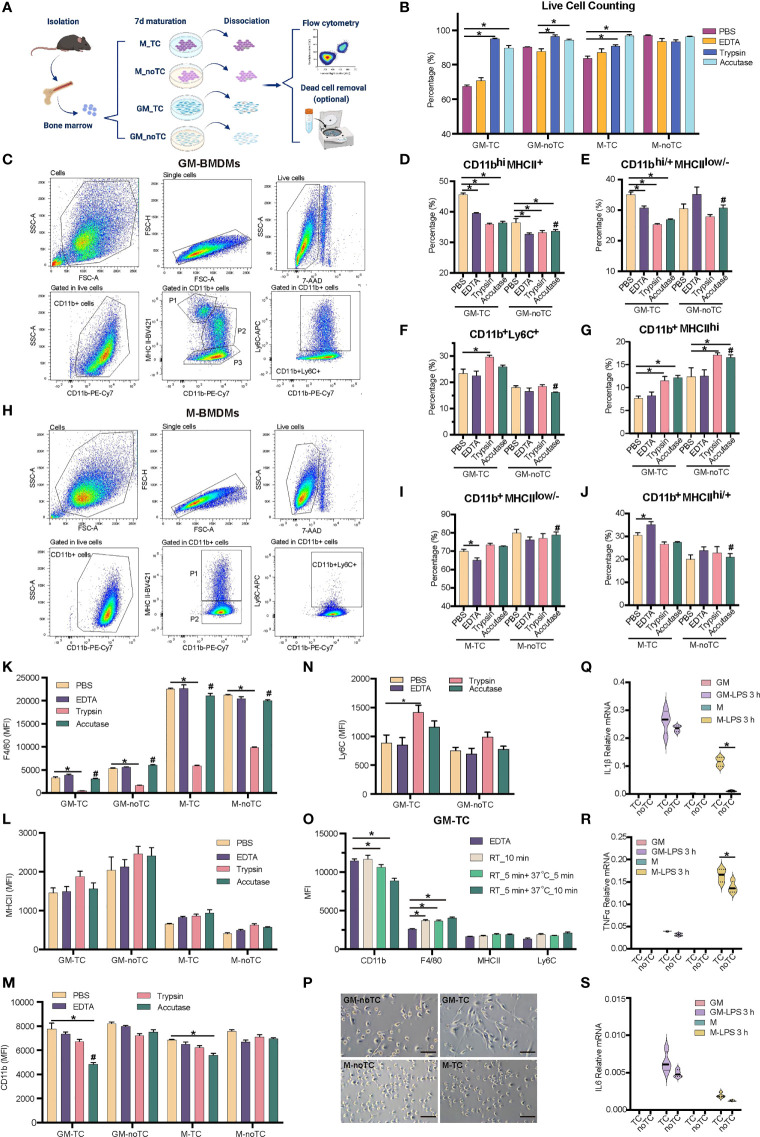
Detaching approaches were performed with two types of BMDMs. **(A)** Schematic diagram of experimental workflow. Post 7 days’ maturation, GM and M were dissociated by enzymic or nonenzymic methods with pipetting or scraping as shown in **Table 1**. The percentage of subpopulations was detected by flow cytometry. Dead cells were further discarded using gradient centrifugation for GM-TC samples when more viable GM-TC cells were needed. **(B)** Percentages of live BMDMs (n = 4 for each group) using different dissociation methods without dead cell removal process. **(C, H)** Gating strategies for GM- **(C)** and M- **(H)** BMDMs cultured on TC dishes and dissociated with accutase followed by necessary mechanical steps in flow cytometry. BMDMs were detached, blocked and stained with the indicated antibodies. Cells were gated for single cells, followed by gating live cells (7AAD-) and CD11b+ populations. The live 7AAD- CD11b+ cells were further analyzed for the specific subpopulations. **(C)** P1: CD11b^+^MHCII^hi^, P2: CD11b^hi^MHCII^+^, P3: CD11b^hi/+^MHCII^low/-^, and CD11b^+^Ly6C^+^ cells; **(H)** P1: CD11b^+^MHCII^hi/+^, P2: CD11b^+^MHCII^low/-^, and CD11b^+^Ly6C^+^ cells. **(D–G)** The percentages of CD11b^hi^MHCII^+^ **(D)**, CD11b^hi/+^MHCII^low/-^ **(E)**, CD11b^+^Ly6C^+^ **(F)** and CD11b^+^MHCII^hi^ **(G)** subpopulations in GM-TC and GM-noTC samples. **(I, J)** The percentages of CD11b^+^MHCII^low/-^ **(I)** and CD11b^+^MHCII^hi/+^ **(J)** subpopulations in M-TC and M-noTC samples. **(K–N)** MFI values of F4/80 **(K)**, MHCII **(L)**, CD11b **(M)** and Ly6C **(N)** in the CD11b+ cells of GM and M. **(O)** MFI values of CD11b, F4/80, MHCII and Ly6C in the CD11b+ cells of GM-TC samples dissociated with EDTA (control group) or accutase at different temperatures and treatment times (n = 4 for each group). RT_10 min means incubation with accutase for 10 min at RT, RT_5 min +37°C_5 min means incubation for 5 min at RT followed by 5 min at 37°C, and RT_5 min+37°C_10 min means incubation for 5 min at RT followed by 10 min at 37°C. **(P)** Cell morphology of GM and M cultured on TC and noTC dishes. Scale bars, 100 μm. **(Q–S)** GM and M cells were treated with LPS (100 ng/ml) for 3 h. Total RNA was prepared and used to determine relative mRNA levels of proinflammatory factors IL1β **(Q)**, TNFα **(R)** and IL6 **(S)**. One-way ANOVA was performed for **(D–G)** and **(I, J)**. Two-way ANOVA was performed for **(B)**, **(K–O)** and **(Q-S)**. For **(B)**, *P* < 0.05 is indicated by * for the comparison of other 3 dissociation methods vs the group with the lowest cell viability in each type of BMDMs. For **(D-G)**, **(I, J)** and **(K–N)**, *P* < 0.05 is indicated by * for the comparison of other 3 dissociation methods vs PBS groups within GM-TC, GM-noTC, M-TC and M-noTC samples, and # for comparison of the accutase dissociated samples between GM-TC vs GM-noTC or M-TC vs M-noTC. For **(O)**, *P* < 0.05 is indicated by * for the comparison of accutase treated samples with EDTA treated ones. For **(Q–S)**, *P* < 0.05 is indicated by * for the comparison of the TC vs noTC samples under the same conditions.

Statistical analyses were performed using Prism version 9. Data were graphically represented as the mean + SD. Statistical significance of differences between indicated samples was determined by paired or unpaired student’s *t*-test or one-way or two-way ANOVA. The Turkey’s multiple comparison test was used for determining statistical significance for one- or two-way ANOVA analysis except **Figures 1Q–S** (two-way ANOVA and Šidák multiple comparison test) and **Figure S1D–G** (paired two-way ANOVA and Šidák multiple comparison test). * and **^#^** p< 0.05 is considered significant.

## Results

### Impact of Detaching Methods on Distinct Macrophage Phenotype and Function

In order to find out the optimal approach for dissociating GM- and M-BMDMs cultured on TC or noTC dishes, four frequently used detaching methods (7), including accutase, trypsin, EDTA and PBS were performed together with mechanically detaching methods (pipetting and scraping) (**Figure 1A**, details in **Table 1**). M-BMDMs cultured on either TC or noTC dishes (M-TC and M-noTC) were easily detached using both enzymatic and non-enzymatic methods with relatively short-term incubation and high cell viability (>85% except for PBS-M-TC) (**Figure 1B**). GM-BMDMs cultured on noTC dishes (GM-noTC) required a relatively longer incubation time than M-BMDMs, but still, maintained an easily detaching status in all tested dissociation methods with high cell viability (>85%). However, GM-BMDMs cultured on TC dishes (GM-TC) were very hard to be detached and incubation with accutase or trypsin followed by pipetting and scraping is the optimal approach to harvest as many cells as possible with relatively high cell viability (**Figure 1B**).

Since cell surface markers are widely used in identifying the macrophage populations, we then investigated whether different detaching methods will influence the protein levels of classical BMDM maturation surface markers such as CD11b, Ly6C, MHCII and F4/80 (4, 30). As shown in **Figure 1C**, GM-BMDMs could be divided into four subpopulations: CD11b^+^MHCII^hi^, CD11b^hi^MHCII^+^, CD11b^hi/+^MHCII^low/-^ and CD11b^+^Ly6C^+^ cells. M-BMDMs were mainly divided into two subpopulations: CD11b^+^MHCII^high/+^ and CD11b^+^MHCII^low/-^ (**Figure 1H**). The percentages of subpopulations in GM-BMDMs differed among different dissociation methods (**Figures 1D–G**). Similarly, the percentages of subpopulations in M-TC but not M-noTC were also interfered by dissociation approaches (**Figures 1I, J**). We further evaluated these markers’ expression levels under various dissociating methods (**Figures 1K–N**). The mean fluorescence intensity (MFI) of F4/80 was remarkably reduced in all trypsin treated samples regardless of the incubation time and temperature used in different cell and dish types (**Figure 1K**). The MFIs of CD11b were interfered only in TC cultured BMDMs to some extent by accutase incubation at RT for 5 min followed by 37°C for 10 min, especially for GM-TC samples (**Figure 1M**). However, when removing the incubation at 37°C but extending the incubation at RT, the MFI of CD11b (RT_10 min group) was comparable to that of control group (**Figure 1O**). Besides, there were no observed changes in the percentages of CD11b^+^MHCII^hi^ and CD11b^hi^MHCII^+^ subpopulations in those groups (**Figure S1A**). There was some moderate alteration in F4/80 levels when comparing EDTA and accutase (RT_10 min) treated groups (**Figure 1O**). The modest changes in the percentages of CD11b^hi/+^MHCII^low/-^ and CD11b^+^Ly6C^+^ subpopulations were also observed in the accutase (RT_10 min) treated samples comparing with EDTA controls (**Figure S1A**), which might be partially due to different cell viabilities of these subpopulations under these detaching treatments. Collectively, these data suggested that accutase is the optimal dissociation approach for all the BMDMs we explored regardless of the growth factor and culture dish types.

To obtain higher cell viability for GM-TC samples, we further performed density gradient centrifugation to remove cell debris and dead cells post their detachment from the dish. As shown in **Figures S1B–C**, cell viability was increased post density gradient centrifugation, especially for EDTA treated GM-TC samples (from 70.77% to 92.32%). Subpopulation distribution and marker gene expression post dead cell removal were similar to the original ones (**Figures S1D–G**). The density centrifugation provided a complementary approach to further enhance the cell viability.

Even though quite similar morphology was observed for GM-TC and GM-noTC, as well as M-TC and M-noTC (**Figure 1P**), the type of culture dishes did have impacts on the percentages of different subpopulations. Post accutase incubation, percentages of all four GM-BMDM and two M-BMDM subpopulations cultured on TC dishes were relatively different comparing with noTC cultured ones (**Figures 1D–J**), especially for CD11b^+^Ly6C^+^ (**Figure 1F**) and CD11b^+^MHCII^hi^ (**Figure 1G**) in GM-BMDMs. Besides, post toll-like receptor 4 (TLR4) agonist LPS treatment (31), the mRNA levels of IL1β, TNFα and IL6 were upregulated in both TC and noTC cultured GM- and M-BMDMs but were less strongly activated in noTC cultured cells (**Figure 1Q–S**). Considering that LPS could reorganize cell actin and influence macrophage adhesion (32), the dissociation efficacies of LPS activated BMDMs by accutase were determined and accutase could also efficiently harvest BMDMs post LPS treatment (**Figure S1H**) despite the fact that LPS treatment changed the distribution of cell subpopulations and marker gene expression in GM- and M-BMDMs (**Figures S1I–R**).

### Transcriptome Profiles of Two Types of BMDMs Cultured on TC and noTC Dishes

**Figure 2:**
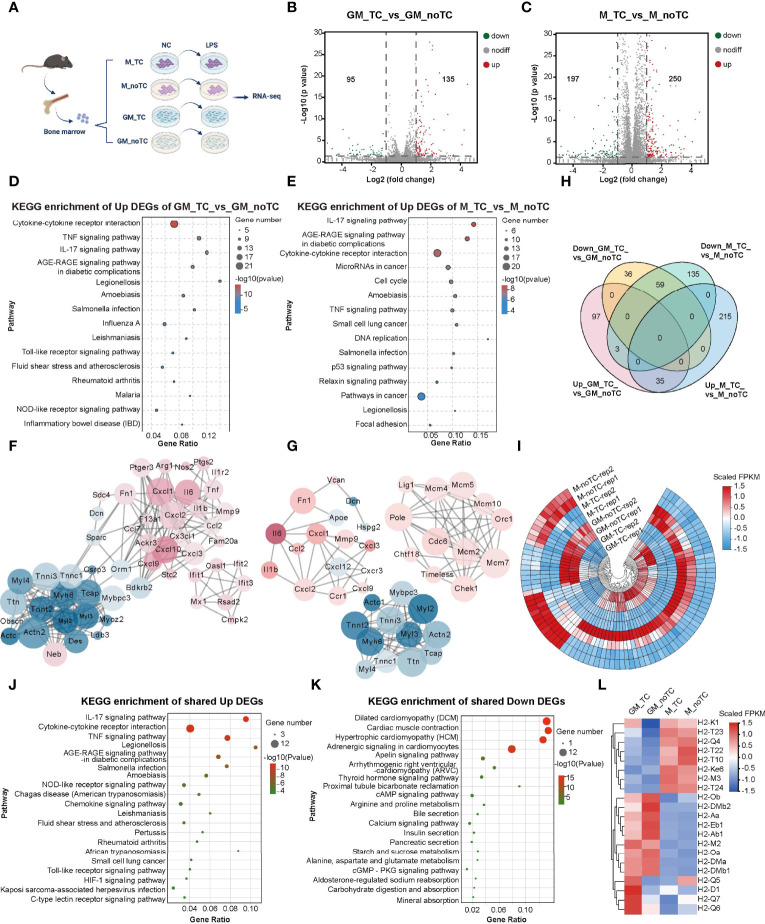
Transcriptome profiling of the impact of different culture conditions on BMDMs at steady state. **(A)** Schematic diagram of experimental workflow. GM and M cells cultured on TC and noTC dishes, in resting and 3 h LPS-stimulated states, were processed for RNA-seq analysis (n= 2 for each group). **(B, C)** The volcano plot of DEGs (fold change (FC)>2 and p<0.05) in GM-TC_vs_GM-noTC groups **(B)** and M-TC_vs_M-noTC groups **(C)**. The grey dots represent genes without significant difference between two groups, the red dots for significantly upregulated DEGs and green dots for significantly downregulated ones. **(D, E)** The top 15 category terms of KEGG analysis of upregulated DEGs in GM-TC_vs_GM-noTC groups **(D)** and M-TC_vs_M-noTC groups **(E)**. **(F, G)** PPI analysis of DEGs in GM-TC_vs_GM-noTC groups **(F)** and M-TC_vs_M-noTC groups **(G)**. Red nodes indicate upregulated DEGs in the noTC group while green ones indicate downregulated ones. The node size represents the connectivity degree. Node color represents the value of LogFC. **(H)** Venn diagram of DEGs among indicated comparison groups. **(I)** Cluster heat maps of the shared 35 upregulated and 59 downregulated DEGs in **(H)**. Rep1 and rep2 represent two biological replicates of samples for RNA-seq analysis. **(J, K)** The top 15 category terms of KEGG analysis of shared upregulated DEGs **(J)** and downregulated DEGs **(K)** in **(I)**. **(L)** Hierarchical cluster analysis for the expression of all MHC genes found in our RNA-seq analysis. For **(I, L)**, color scale represents scaled FPKM values normalized using z-score approach.

To further investigate the influence of different culture conditions on macrophage gene expression and function, we performed RNA-seq analysis of GM-BMDMs (GM) and M-BMDMs (M) on TC and noTC dishes at resting/steady state or upon LPS challenge (GSE198821) (**Figure 2A**). At steady state, there were 135 significantly upregulated and 95 significantly downregulated differentially expressed genes (DEGs) in GM-noTC cells comparing with GM-TC counterparts (**Figure 2B**), whereas 250 upregulated and 197 downregulated DEGs in M-noTC comparing with M-TC cells (**Figure 2C**). KEGG functional analysis of those 135 upregulated DEGs in GM-noTC group identified a variety of proinflammatory pathways ranked in top 15 terms, including cytokine-cytokine receptor interaction, TNF signaling pathway and IL-17 signaling pathway, as well as inflammatory diseases, such as inflammatory bowel disease (**Figure 2D**). Similarly, GO (**Figure S2A**) and Reactome (**Figure S2B**) enrichment analysis also pointed to the inflammation-associated diseases and functions. We also performed protein-protein interaction (PPI) analysis and found that a bunch of upregulated proinflammatory factors including CXCL1, CXCL2, CXCL10, IL6, MMP9, TNF, FN1, CX3CL1 (in red color) were cored within the network (**Figure 2F**). Similarly, KEGG functional analysis of 250 upregulated DEGs in M-noTC comparing with M-TC revealed that the top 15 upregulated biological pathways ranked by significance also included inflammation-related pathways, such as cytokine-cytokine receptor interaction, TNF signaling pathway and IL-17 signaling pathway, as well as cell proliferation and differentiation related pathways, including cell cycle and DNA replication (**Figure 2E**). GO and Reactome enrichment analysis also confirmed that those upregulated DEGs were enriched in inflammation and cell cycle-associated terms (**Figures S2C–D**). In the PPI network, multiple inflammatory factors (IL1β, IL6, CXCL1, CXCL2 and FN1) and cell cycle/DNA replication related genes (LIG1, POLE, MCM4, MCM5, MCM10, ORC1 and CDC6) were identified as the upregulated hub genes in M-noTC group (**Figure 2G**). Taken together, these results suggested that proinflammatory signaling was enriched in noTC cultured BMDMs regardless of polarizing growth factors. Meanwhile, in the PPI network of DEGs of both GM-TC_vs_GM-noTC and M-TC_vs_M-noTC, actin cytoskeleton organization related genes such as ACTC1, ACTN2, CSRP3, MYBPC3 and MYL2 were identified as the downregulated hub genes (**Figures 2F, G**).

There are 59 downregulated and 35 upregulated DEGs shared in GM-BMDMs and M-BMDMs when comparing no-TC samples with their TC counterparts (**Figures 2H, I**). KEGG analysis of these shared 35 upregulated DEGs uncovered that IL-17 signaling pathway, cytokine-cytokine receptor interaction and TNF signaling pathway were enriched in top 15 KEGG pathways (**Figure 2J**). The top 15 KEGG terms of shared downregulated DEGs were multiple metabolic pathways and the related cAMP/cGMP signaling (**Figure 2K**). These results confirmed that culturing on noTC dish could enhance a variety of proinflammatory signal pathways in both GM- and M-BMDMs.

Besides, gene expression levels of MHCI and MHCII were determined. In consistence with flow cytometry data shown in **Figure 1L**, the mRNA levels of MHCII family including H2-Ob, H2-Aa, H2-Eb1 and H2-Ab1 were higher in GM-noTC than GM-TC ones (**Figure 2L**).

### Comparison of LPS Induced Inflammatory Responses in Two Types of BMDMs Cultured on TC and noTC Dishes

Next, we evaluated the LPS induced transcriptome changes of BMDMs cultured in different conditions. As shown in **Figure S3A**, hundreds of genes were significantly influenced following LPS stimulation. There were 1195 upregulated and 2495 downregulated DEGs in GM-TC samples upon LPS challenge (GM-TC_vs_GM-TC-LPS) (**Figure S3B**) and 1258 upregulated and 3874 downregulated DEGs in M-TC-LPS comparing with M-TC (**Figure S3C**). Using IPA analysis (33), significant DEGs related diseases and functions were summarized. In both LPS treated GM-TC and M-TC samples, a variety of inflammation-associated terms were predicted to be activated, including IL6, TNF, IL1β, IFNG (IFNγ), DOCK8 and IL12β (**Figures S3D, E**). KEGG functional enrichment analysis also indicated that LPS induced various proinflammatory signaling activities in both GM-TC and M-TC groups (**Figures S3F–G**).

**Figure 3:**
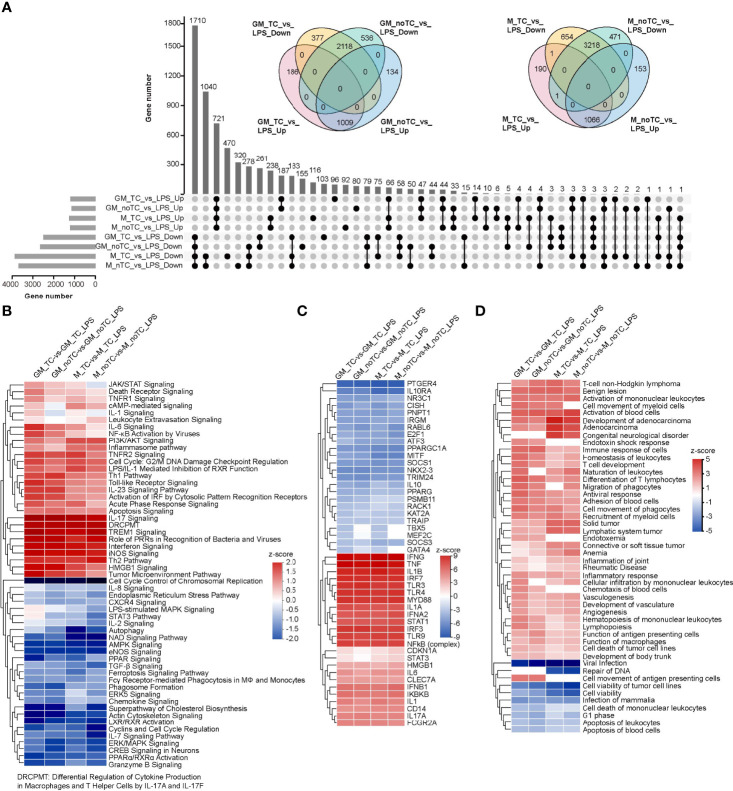
Comparative analysis of LPS induced gene and functional changes of two types of BMDMs cultured on TC and noTC dishes using IPA software. **(A)** The Upset plot showed the number of shared DEGs of the indicated comparison of BMDMs at resting state and upon LPS challenge. Venn diagram of DEGs among the indicated comparison groups were embedded in the upper panels. **(B-D)** Heatmap of the representative canonical pathways **(B)**, upstream regulators **(C)** and disease and function **(D)** between GM-TC_vs_GM-TC-LPS groups, GM-noTC_vs_GM-noTC-LPS groups, M-TC_vs_M-TC-LPS groups and M-noTC_vs_M-noTC-LPS groups. Positive z scores indicate an increase whereas negative ones indicate a reduction in LPS treated groups calculated using IPA software. n= 2 for each group.

The DEGs of each comparison were analyzed to find LPS induced common gene expression changes (**Figure 3A**). There were 1009 upregulated and 2118 downregulated DEGs shared in the comparison of GM-TC_vs_GM-TC-LPS and GM-noTC_vs_GM-noTC-LPS. A total of 1066 upregulated and 3218 downregulated DEGs were shared in M-TC_vs_M-TC-LPS and M-noTC_vs_M-noTC-LPS. High similarities were observed and among them 721 upregulated and 1710 downregulated DEGs were shared in all the four comparison groups regardless of growth factors and culture conditions. To deeply elaborate the influence of LPS-stimulated inflammatory responses in BMDMs under different culture conditions, we further performed comparison analysis using IPA software. There were high similarities in terms of the representative signaling pathways, upstream regulators and disease/functions across all four comparing groups (**Figures 3B–D**). In canonical pathway comparison, proinflammatory signaling pathways, such as NFκB signaling, toll-like receptor signaling, interferon signaling and IL-17 signaling were strongly activated post LPS challenge in all the comparisons (GM-TC_vs_GM-TC-LPS, GM-noTC_vs_GM-noTC-LPS, M-TC_vs_M-TC-LPS and M-noTC_vs_M-noTC-LPS) (**Figure 3B**). Besides, immunosuppressive and cell cycle related signaling pathways post LPS challenge were commonly inhibited, including PPAR signaling, TGF-β signaling, actin cytoskeleton signaling, cyclins and cell cycle regulation and cell cycle control of chromosomal replication. Consistently, upstream regulatory factors including IFNG (IFNγ), TNF, IL1β, TLR3, TLR4, MYD88, NFκB (complex) were significantly upregulated, while PTGER4, IL10R4, IL10, PPARGC1A, MITF, SOCS1, NKX2-3 and TRIM24 were significantly downregulated in all the comparisons (**Figure 3C**). In the biological function and disease comparisons, immune cell adhesion, migration, mobility and inflammatory responses were upregulated after LPS stimulation in both GM-BMDMs and M-BMDMs cultured on both TC and noTC dishes (**Figure 3D**). These data sugggested that LPS could induce relatively comparable inflammatory responses in both TC and noTC cultured BMDMs.

### Comprehensive Analysis of the Influence of Culture Dish Surface on Two Types of BMDMs Upon LPS Challenge

**Figure 4:**
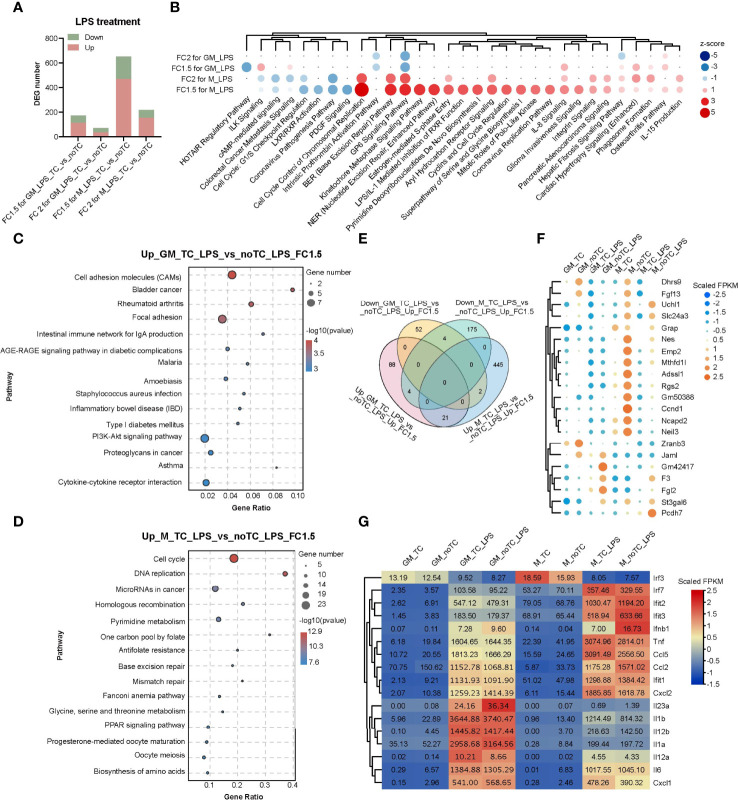
Transcriptome analysis of the influence of different culture dishes on BMDMs under LPS treatment. **(A)** Bar chart of DEGs among different groups. Fold change > 2 or 1.5 and p-value < 0.05 (n= 2 for each group). **(B)** The top canonical pathway comparison analysis of the DEGs (FC > 2 or 1.5 and p < 0.05) in **(A)**. FC2 for GM_LPS means GM-TC-LPS vs GM-noTC-LPS (FC > 2 and p < 0.05) groups. Others were named in the same way. Positive z scores indicate an increase whereas negative ones indicate a reduction in LPS treated noTC cultured groups calculated using IPA software. **(C, D)** The top 15 category terms of KEGG analysis of upregulated DEGs in GM-TC-LPS_vs_GM-noTC-LPS groups **(C)** and M-TC-LPS_vs_M-noTC-LPS groups **(D)**. **(E)** Venn diagram of DEGs among indicated comparison groups. **(F)** Cluster heatmap analysis of the shared 21 upregulated DEGs in **(E)**. **(G)** Heat map analysis of classical proinflammatory factors in LPS-related pathways of each group. For **(F, G)**, color scale represents scaled FPKM values normalized using z-score approach.

Comparing with the gene expression profiles at steady state (**Figures 2B,C**), there were fewer DEGs (fold change > 2, p < 0.05) in the comparison of noTC and TC cultured cells upon LPS treatment (**Figure 4A**), especially for GM-TC-LPS_vs_GM-noTC-LPS comparison (36 upregulated and 36 downregulated DEGs), which indicated that the influence of culture dish surface on gene expression was weakened upon LPS challenge. To better elucidate the possible impact of different culture conditions on macrophage functions (**Figure 4B**), DEGs with fold change > 1.5 (p < 0.05) were extracted from RNA-seq data of LPS challenged different types of BMDMs (**Figure 4A**). There are 113 upregulated and 58 downregulated DEGs in GM-noTC-LPS comparing with GM-TC-LPS, while 468 upregulated and 183 downregulated DEGs in M-noTC-LPS comparing with M-TC-LPS group. KEGG enrichment analysis of the upregulated DEGs of GM-TC-LPS_vs_GM-noTC-LPS indicated that inflammatory signaling pathways and diseases, as well as cell adhesion associated signaling, were ranked at top 15 KEGG terms (**Figure 4C**). For LPS stimulated M-BMDMs, the top 15 upregulated KEGG pathways included cell cycle, DNA replication, mismatch repair and several metabolic pathways (**Figure 4D**). These data suggested that culture condition interfered signaling pathways were different in GM-CSF and M-CSF differentiated BMDMs post LPS challenge, pointing to the diverse impacts of culture dish on gene expression at inflammatory state. Furthermore, we compared the DEGs in LPS challenged GM-BMDMs and M-BMDMs (**Figure 4E**). In consistent with the above functional analysis, there were only 21 upregulated and 4 downregulated shared genes in the comparison, suggesting the dissimilarity of culture dish-dependent responses in GM-BMDMs and M-BMDMs post LPS treatment. Among the 21 shared upregulated DEGs, Mthfd1l, Adssl1 and St3gal6, which were related to anabolism and Dhrs9, which was related to retinol metabolism were included (**Figure 4F**). Under both TC and noTC culture conditions, LPS induced TLR4-MyD88 and TRIF/IRF dependent signaling pathways (30) were upregulated in GM-BMDMs and M-BMDMs (**Figure 4G**). The expression levels of TRIF-dependent Ifnb1, Ccl2 and interferon stimulating genes (ISGs) such as Ifit1, Ifit2, Ifit3 were higher whereas Irf7, Tnf and Ccl5 were lower in M-noTC-LPS comparing with M-TC-LPS. Meanwhile, the expression of MyD88 dependent signalings such as IL23a (IL23α), IL1b (IL1β) and IL1a (IL1α) were upregulated in GM-noTC-LPS comparing with GM-TC-LPS. These data indicated that under the LPS challenge, the gene expression changes caused by culture dishes were largely dependent on differentiation growth factors.

### IPA Comparison of the Influence of Culture Dish Surface on BMDMs at Steady State and Post LPS Stimulation

**Figure 5:**
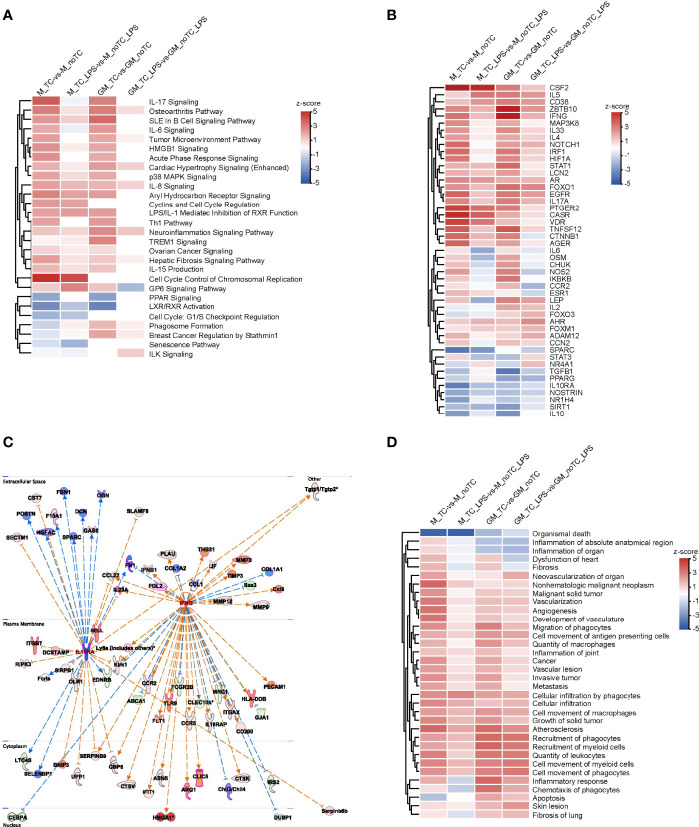
The effects of different culture dishes under the resting and inflammatory states for BMDMs by IPA comparison. **(A, B, D)** Canonical pathway comparison **(A)**, Upstream regulator comparison **(B)**, and Disease and biological function comparison **(D)** were conducted by comparing significant differences between M-TC vs M-noTC groups, M-TC-LPS vs M-noTC-LPS groups, GM-TC vs GM-noTC groups and GM-TC-LPS vs GM-noTC-LPS groups. Positive z scores indicate an increase whereas negative ones indicate a reduction in the noTC cultured BMDMs calculated using IPA software. **(C)** These selected upstream regulator gene networks of the anti-inflammatory factor (IL10R) and proinflammatory factor (IFNG) and relationship for the related DEGs between the GM-TC-LPS vs GM-noTC-LPS groups and M-TC-LPS vs M-noTC-LPS groups. n= 2 for each group. The edges connecting the nodes are colored orange when upstream regulators have activating effects on their target genes and blue when they have inhibiting effects.

In order to comprehensively illustrate the effects of dish surface on the gene expression profiles of BMDMs in both steady state and LPS-stimulated inflammatory state, we utilized IPA software to conduct further comparative analysis (**Figure 5**). In canonical pathway analysis, most inflammation-associated signal pathways were upregulated in noTC cultured BMDMs, at both basal level and after LPS stimulation, including HMGB1 signaling, osteoarthritis pathway, p38 MAPK signaling, IL8 signaling and TREM1 signaling. The anti-inflammatory function related LXR/RXR activation and cell cycle related G1/S checkpoint regulation were downregulated (**Figure 5A**). In the upstream regulator comparison, certain regulatory factors were intensively upregulated across all the comparison groups, including CSF2, IL5, CD38, ZBTB10, IFNG, MAP3K8, IL33, IL4, NOTCH1, IRF1, HIF1A, STAT1, LCN2, AR, FOXO1, EGFR, IL17A, PTGER2, CASR, VDR, TNFSF12 and AGER, while the downregulated factors included IL10RA, NOSTRIN, SIRT1 and IL10. However, IL6, OSM, CHUK, NOS2, IKBKB and CCR2 were clustered to be upregulated at the steady state but downregulated post LPS stimulation in both GM-BMDMs and M-BMDMs cultured on noTC dishes comparing with those on TC dishes (**Figure 5B**). The activated and inhibited gene networks mastered by proinflammatory IFNG (IFNγ) and anti-inflammatory IL10RA were shown in **Figure 5C**. The adhesion related gene ITGB7 was connected with IL10RA, whereas migration related gene MMP9 and adhesion related genes (COL1A1, PECAM1 and HLA-DOB) were connected with IFNG. In the biological function and disease comparison, the terms related to cell adhesion, such as cell infiltration by phagocytes, cell movement of macrophages, and recruitment of phagocytes, were all upregulated in noTC cultured BMDMs under both steady state and LPS challenge (**Figure 5D**). These data suggested that under resting and stimulated conditions, there were some shared upregulated canonical pathways, upstream regulators and disease/functions, mainly inflammation associated ones, regardless of differentiation and polarizing growth factors. However, dissimilarity of the influence of culture conditions in GM-BMDMs and M-BMDMs also existed, in which some of them relied on cell states (LPS challenge or not) and others depended on growth factors (GM-CSF and M-CSF).

### Cell Adhesion, Cell Cycle and Cytokines Were Centered on the Similarities of BMDMs Under Different Culture Conditions and Cell Status

**Figure 6:**
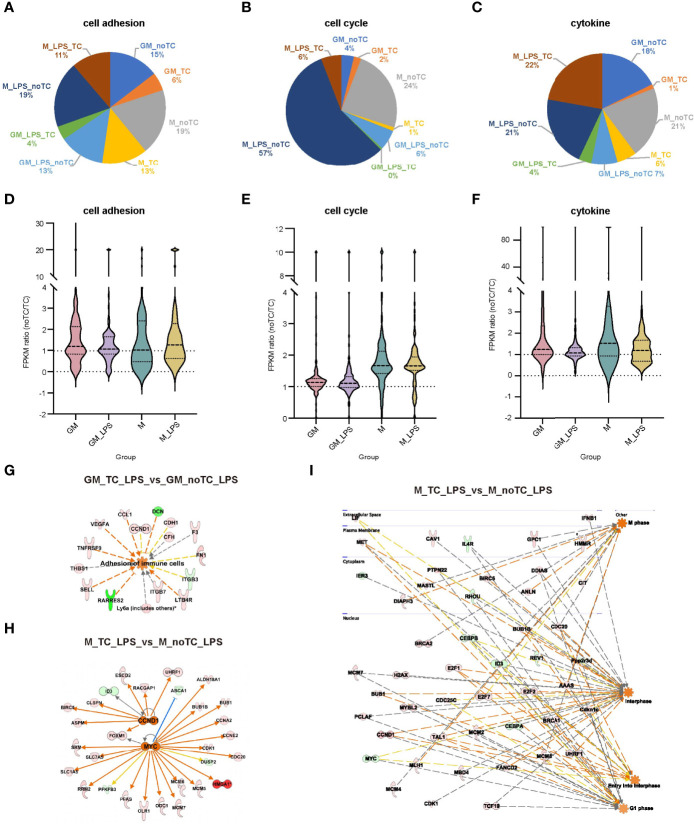
The gene expression profiles related to cell adhesion, cell cycle and cytokines exhibited similar trends in BMDMs. **(A–C)** The gene number distribution of DEG related to cell adhesion **(A)**, cell cycle **(B)** and cytokine **(C)** in the comparison of noTC and TC groups of GM and M at resting state and post LPS challenge. **(D–F)** The fpkm ratio (noTC/TC) of total enriched DEGs of the indicated functional terms of GM and M at both resting state and post LPS challenge. **(G)** The predicted upregulated adhesion related functional gene network of “adhesion of immune cells” using IPA software. Orange indicates upregulated upstream regulators/function in GM-noTC-LPS comparing with GM-TC-LPS. Green indicates downregulated DEGs while red indicates upregulated DEGs in GM-noTC-LPS. **(H–I)** The predicted upregulated cell cycle related upstream regulators including CCND1 and MYC **(H)** and functions such as M phase, interphase, entry into interphase and G1 phase **(I)** and their DEG network diagram in M-TC-LPS_vs_M-noTC-LPS comparison using IPA software. Orange indicates upregulated upstream regulators/function in M-noTC-LPS comparing with M-TC-LPS. Green indicates downregulated DEGs while red indicates upregulated DEGs in M-noTC-LPS. The edges connecting the nodes are colored orange when upstream regulators have activating effects on their target genes and blue when they have inhibiting effects.

Although relatively high similarities of gene expression patterns existed when comparing no-TC-BMDMs with their counterparts, there were also certain differences. Based on the above functional analysis, cytokine response, cell adhesion and cell cycle associated signaling pathways were the most prominent differences. For noTC cultured BMDMs, both at steady state and after LPS challenge (**Figure 6A**), cell adhesion related gene numbers were higher in GM-noTC group comparing with GM-TC group, while no obvious preference was observed for M-TC and M-noTC groups. On the contrary, M-noTC (24%) and M-noTC-LPS (57%) had the majority of cell cycle related genes, which was higher than M-TC and M-TC-LPS groups (**Figure 6B**). Similar pattern was noticed in GM-noTC samples but cell cycle related genes in them were too few to draw conclusion. Consistent with the shared upregulated “cytokine-cytokine receptor interaction” function in noTC samples at steady state, cytokine related gene numbers in GM-noTC and M-noTC groups were higher than GM-TC and M-TC groups at steady state (**Figure 6C**). Besides, the overall expression levels of genes related to cell cycle, cell adhesion and cytokine production were also higher in the indicated noTC cultured BMDMs (**Figures 6D-F**). Consistently, immune cell adhesion associated gene network was activated in GM_noTC_LPS group comparing with GM_TC_LPS group in IPA analysis (**Figure 6G**). Based on DEGs of M_TC_LPS_vs_M_noTC_LPS, the cell cycle related gene network including the upstream regulators CCND1 and MYC and connected DEGs (**Figure 6H**), as well as the indicated cell phases and connected DEGs (**Figure 6I**), were enriched in M_noTC_LPS group. In conclusion, at both steady and inflammatory states, noTC cultured GM-BMDMs were biased to upregulate the function of cell adhesion and M-BMDMs were prone to enhance the cell cycle progression, pointing to the differential influences of culture dish surfaces on the functions of different macrophage populations.

## Discussion

Establishing the optimal culture conditions and cell detaching methods is indispensable for macrophage phenotypic and functional studies. Several reports compared different dissociation methods in M-CSF primed monocyte-derived macrophages challenged with or without IL4 or LPS+IFNγ (7, 8). The influence of different culture conditions (collagen coated 2D surfaces vs collagen 3D scaffold) on certain macrophage populations was also discussed (34, 35). However, the detailed elucidation of the influence of these two aspects, especially TC and noTC culture dishes, to macrophage gene expression and function remains to be determined. In the present study, we compared different detaching methods and found that accutase was the optimal approach for the detachment of M-CSF and GM-CSF differentiated BMDMs with high cell viability and intact cell surface markers. For GM-BMDMs cultured on TC dishes which have strongest adherent capacity, accutase digestion followed by removing dead nonfunctioning cells with a density gradient centrifugation could further enhance the percentage of viable cells. In addition, we performed RNA-seq analysis of GM-BMDMs and M-BMDMs cultured on TC and noTC dishes with or without LPS challenge. Bioinformatic analysis of gene expression patterns at the steady state identified the proinflammatory tendency of BMDMs grown on noTC dishes. Furthermore, the similarity and dissimilarity of the gene expression profiles with different dish surfaces and cell states (steady state and LPS challenge) were also identified. The culture dish-dependent and -independent functions of GM-BMDMs and M-BMDMs were elaborated. Among them, cell adhesion, cytokine and cell cycle associated terms were centered in culture dish-dependent changes.

Preparation of single cell suspension with high cell viability was pivotal to study macrophage functions *in vitro* and to harvest high-quality CAR-engineered macrophage (CAR-M) for disease treatment. The current study compared the efficiency of four different detaching approaches for GM-BMDMs and M-BMDMs grown on TC and noTC dishes with detailed processing protocols. Accutase was suggested as the optimal detaching method and if necessary, density gradient centrifugation to remove cell debris and dead cells was suggested to enrich viable cells. Besides, RNA-seq analyses were performed to explore the gene expression changes caused by culture dish surfaces with different BMDMs under resting and LPS treatment. The noTC-BMDMs exhibited more proinflammatory characters, representing the culture dish-dependent effects. In terms of LPS responsiveness, TC cultured BMDMs did not differ a lot from noTC cultured ones. However, some cell functions such as cell adhesion and cell cycle were interfered, partially dependent on differentiation growth factors (GM-CSF and M-CSF). Therefore, more attentions need to be paid for culture dish selection when certain macrophage functions are studied. Our current study provided the comprehensive and valuable reference information for the related macrophage studies.

Macrophages are widely distributed in tissues, body cavities and mucosal surfaces (36). The surrounding microenvironment is important for cell lineage growth and adequate reconstitution of microenvironment for culturing macrophage *in vitro* is necessary to mimic *in vivo* macrophage behaviors and functions. The mechanical properties of the environment, especially stiffness, are considered as the important parameter in achieving appropriate physiological functions of macrophages (37). Most organs and biological tissues in the body are soft viscoelastic materials with elastic moduli ranging from 1.0×10^2^ Pa in brain to 1.0×10^5^ Pa within soft cartilage. The tissue stiffness surrounding macrophages *in vivo* has certain impacts on their maturation/polarization (38). The TC dishes commonly used for *in vitro* cell culture have a modulus of about 1.0×10^9^ Pa while the modulus of noTC dishes is between 3.0-3.6×10^9^ Pa. The influence of TC and noTC dishes on the cultured BMDMs might partially result from the difference of the stiffness (3-fold difference). Nowadays, not only TC and noTC dishes with high stiffness are commonly used for 2D cell culture, but also the materials with low stiffness such as type I collagen, Matrigel and polyacrylamide gel are also employed to mimic 3D tissue environment for macrophage maturation/polarization (39–41). It has been reported that the 3D environments with different stiffness have great influences on the polarization of macrophages (37, 42). Specifically, low stiffness matrix promoted macrophages transforming into classically activated proinflammatory type M1, while moderate stiffness matrix is in favor of the alternatively activated anti-inflammatory type M2 (34, 35, 43). Therefore, we could speculate that stiffness strength could be tightly correlated with gene expression changes we observed, which could also be used to predict macrophage behaviors *in vivo*. However, this hypothesis needs to be further verified both *in vitro* and *in vivo*.

In summary, we performed systematic evaluation of four commonly used detaching methods for GM-CSF and M-CSF primed BMDMs cultured on TC and noTC dishes treated with or without LPS. High-throughput RNA-seq analysis was utilized to comprehensively analyze the influence of culture dish surfaces on macrophage gene expression profiles and functions. The optimal processing protocol was given and the culture dish-dependent and -independent effects were elucidated to help the selection of culture dishes for studies in different macrophage populations.

## Data Availability Statement

The datasets presented in this study can be found in online repositories. The name of the repository and accession number can be found below: National Center for Biotechnology Information (NCBI) Gene Expression Omnibus (GEO), https://www.ncbi.nlm.nih.gov/geo/, GSE198821.

## Ethics Statement

The animal study was reviewed and approved by the Committee of Experimental Animals of School of Medicine and Pharmacy, Ocean University of China (OUCSMP-20181002).

## Author Contributions

All authors participated in interpreting the whole data. CZ designed and supervised the study; QS, YZ, YX, QZ, ZW, LZ, and LW performed and evaluated individual experiments; QS and YZ performed bioinformatical analyses; QS, YZ, MZ, SL, WP, XL, and CZ wrote the manuscript with the contributions from all the authors. All authors contributed to the article and approved the submitted version.

## Funding

This work was supported by Major Program of National Natural Science Foundation of China (81991525) and Key R&D Program of Shandong Province (2020CXGC010503).

## Conflict of Interest

The authors declare that the research was conducted in the absence of any commercial or financial relationships that could be construed as a potential conflict of interest.

## Publisher’s Note

All claims expressed in this article are solely those of the authors and do not necessarily represent those of their affiliated organizations, or those of the publisher, the editors and the reviewers. Any product that may be evaluated in this article, or claim that may be made by its manufacturer, is not guaranteed or endorsed by the publisher.
