# Supplementary material for: The Culture Dish Surface Influences the Phenotype and Dissociation Strategy in Distinct Mouse Macrophage Populations
Source: Front Immunol. 2022 Jul 6;13:920232. doi: 10.3389/fimmu.2022.920232 (PMC9299442; doi:10.3389/fimmu.2022.920232)
Supplement: Supplementary file 1 [file DataSheet_1.zip › Supplementary material/Supplementary material.docx]

Supplementary Material

# Figure legends

**Figure S1. The influence of gradient centrifugation and LPS treatment on cell distribution and gene expression of BMDMs.**

(A) The percentages of CD11b^+^Ly6C^+^, CD11b^hi^MHCII^+^, CD11b^hi/+^MHCII^low/-^, and CD11b^+^MHCII^hi^ subpopulations dissociated by EDTA (control group) or accutase at different temperatures and treatment time in the GM-TC samples (n = 4 for each group). RT_10 min means incubation with accutase for 10 min at RT, RT_5 min +37°C_5 min means incubation for 5 min at RT followed by 5 min at 37°C, and RT_5 min+37°C_10 min means incubation for 5 min at RT followed by 10 min at 37°C. Unpaired two-way ANOVA analysis was performed. (B-C) The live cell percentages before and after gradient centrifugation to remove dead cells (n = 3 for each group). Paired student’s t test. (D-G) The percentages of CD11b^+^Ly6C^+^, CD11b^hi^MHCII^+^, CD11b^hi/+^MHCII^low/-^ and CD11b^+^MHCII^hi^ subpopulations in the GM-TC samples dissociated by EDTA (D) or accutase (F) before and after gradient centrifugation. MFI values of F4/80, MHCII, CD11b and Ly6C in the GM-TC samples dissociated by EDTA (E) or accutase (G) before and after gradient centrifugation. Paired two-way ANOVA analysis. (H) The live cell percentages of GM-TC, GM-noTC and M-TC cells at resting state (NC) and post 3 h LPS treatment (LPS) dissociated by accutase followed by mechanical steps (n = 4 for each group). Unpaired one-way ANOVA analysis. (I-N) The percentages of CD11b^hi^MHCII^+^ (I), CD11b^hi/+^MHCII^low/-^ (J), CD11b^+^Ly6C^+^ (K) and CD11b^+^MHCII^hi^ (L) subpopulations in GM-TC samples and CD11b^+^MHCII^low/-^ (M) and CD11b^+^MHCII^hi/+^ (N) subpopulations in M-TC post LPS challenge. Unpaired student’s t test. (O-R) MFI values of F4/80 (O), MHCII (P), CD11b (Q) and Ly6C (R) in the CD11b+ populations of GM-TC and M-TC samples. Unpaired one-way ANOVA analysis. * p< 0.05 is considered significant.

**Figure S2. Functional enrichment analysis of DEGs between TC and noTC cultured BMDMs at steady state.**

(A) and (C). GO enrichment analysis of the upregulated DEGs in GM-TC vs GM-noTC comparison (A) and M-TC vs M-noTC comparison (C). (B) and (D). Reactome analysis of upregulated DEGs in GM-TC vs GM-noTC comparison (B) and M-TC vs M-noTC comparison (D).

**Figure S3. Transcriptome analysis of LPS induced-proinflammatory responses for BMDMs cultured on TC dishes.**

(A) Bar chart of DEGs among different groups. Fold change > 2 and p < 0.05. (B-C) The volcano plot of DEGs in GM-TC vs GM-TC-LPS groups (B) and M-TC vs M-TC-LPS groups (C). (D-E) The graphical summary of significant changes in GM-TC vs GM-TC-LPS groups (D) and M-TC vs M-TC-LPS groups (E) by IPA analysis. Symbols of target cytokines, canonical signaling and immune cell regulation in red indicate a predicted increase or activation. (F-G) The top 20 category terms of KEGG analysis of upregulated DEGs in GM-TC vs GM-TC-LPS groups (F) and M-TC vs M-TC-LPS groups (G).
