# Supplementary figures and images for: The Culture Dish Surface Influences the Phenotype and Dissociation Strategy in Distinct Mouse Macrophage Populations
Source: Front Immunol. 2022 Jul 6;13:920232. doi: 10.3389/fimmu.2022.920232 (PMC9299442; doi:10.3389/fimmu.2022.920232)

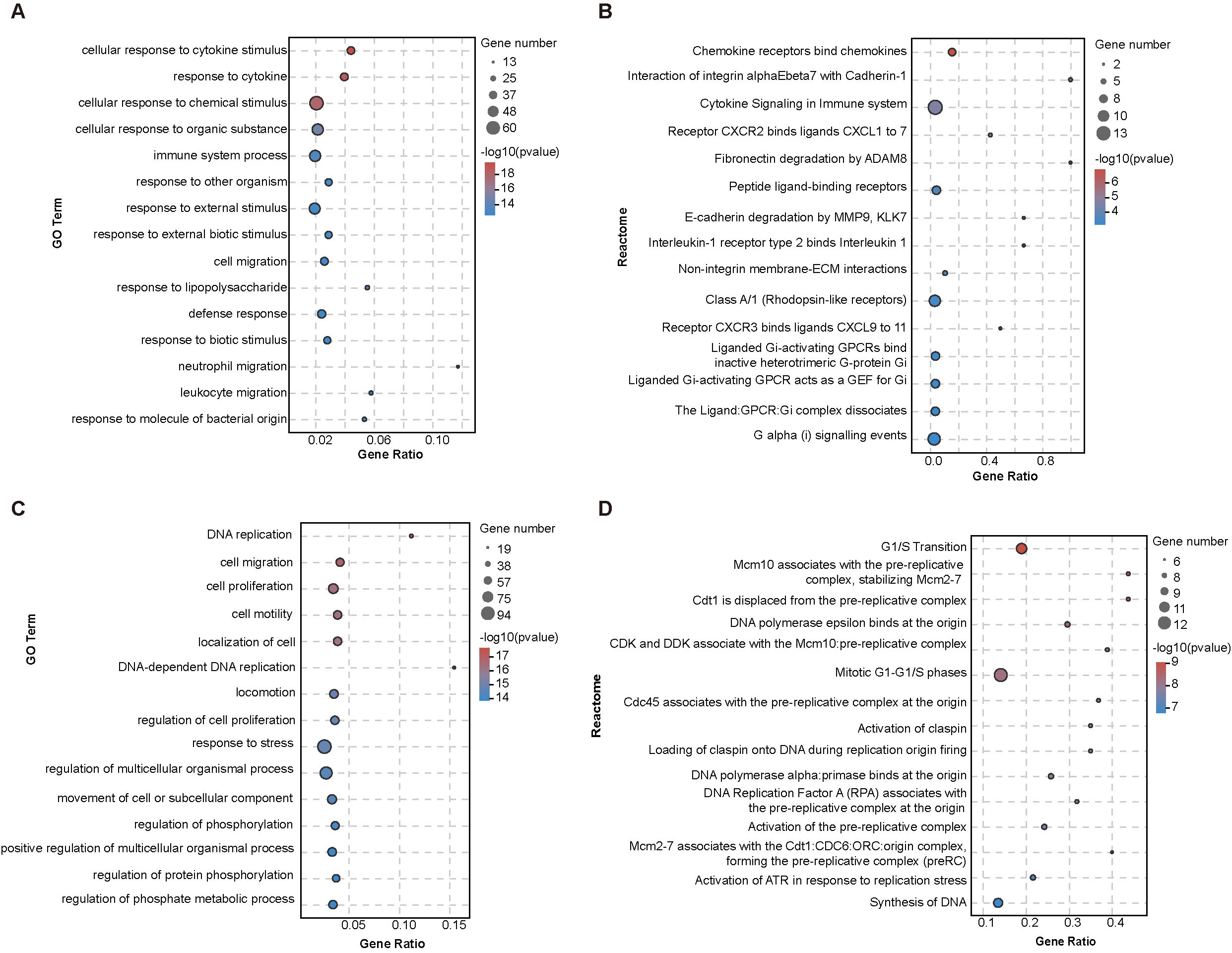

Supplement: Supplementary file 1 [file DataSheet_1.zip › Supplementary material/figure-s2.jpg]

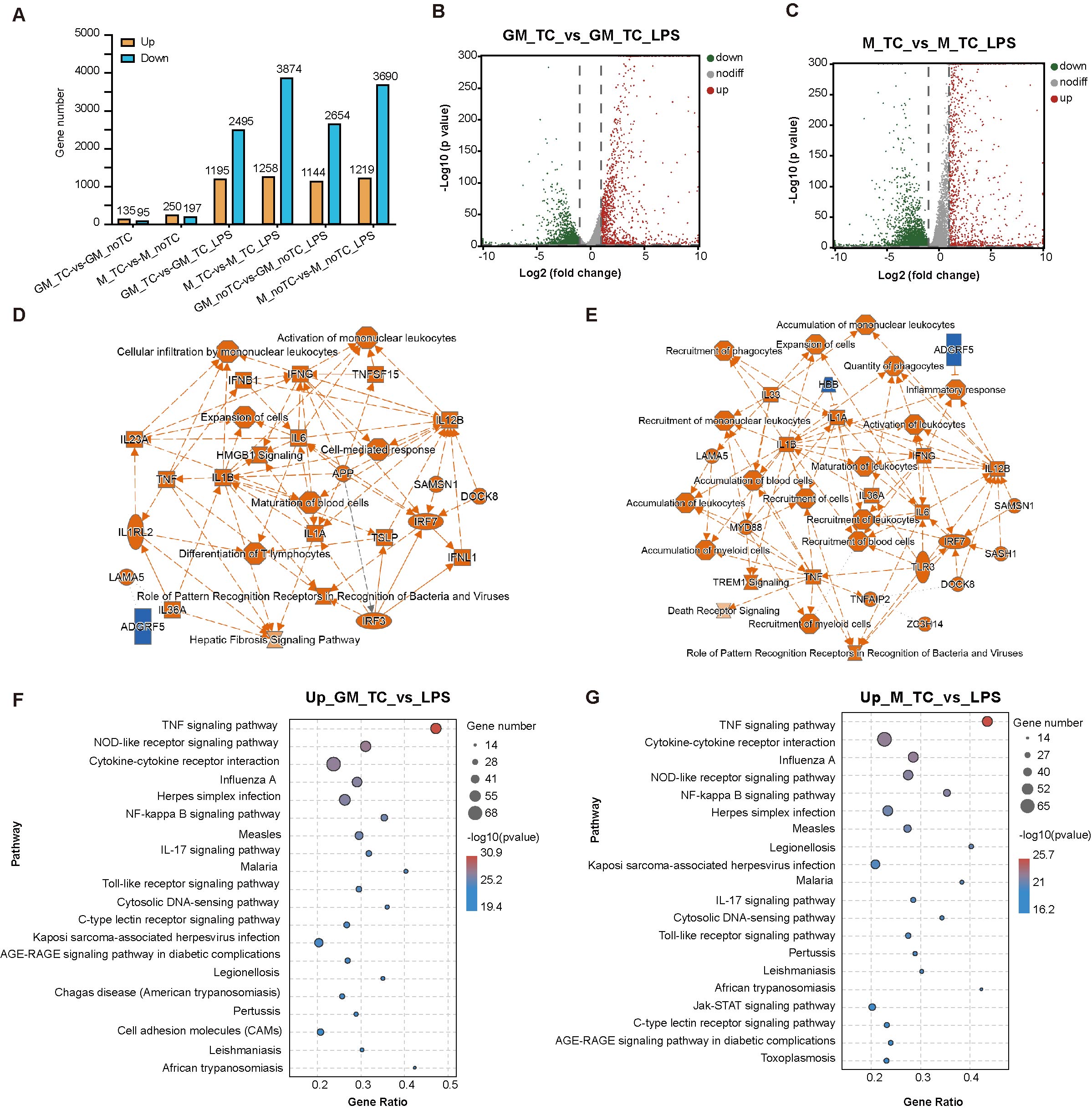

Supplement: Supplementary file 1 [file DataSheet_1.zip › Supplementary material/figure-s3.jpg]

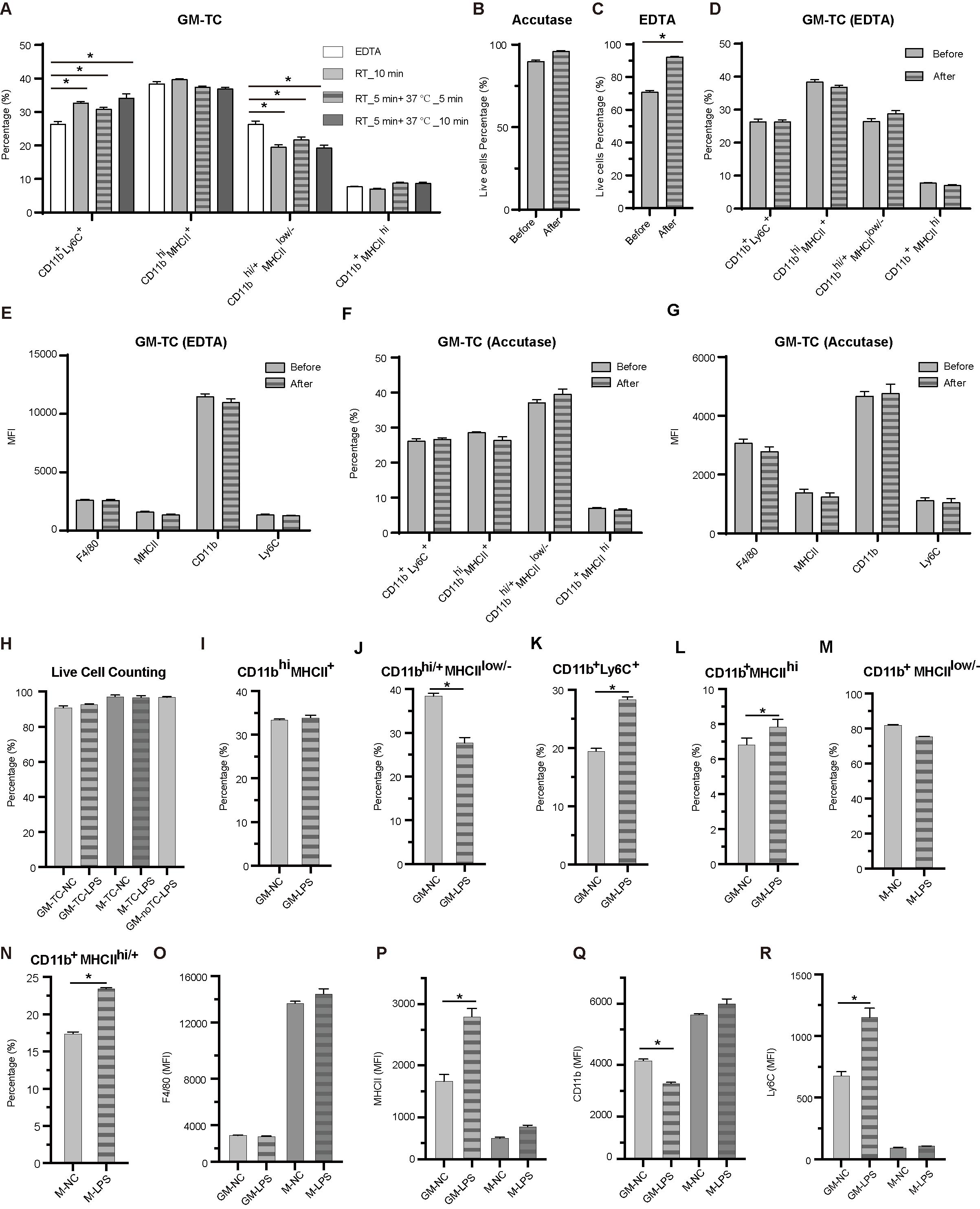

Supplement: Supplementary file 1 [file DataSheet_1.zip › Supplementary material/Figure-s1.jpg]
